# Post-Induction Treatment for Acute Myeloid Leukemia: Something Change?

**DOI:** 10.1007/s11912-021-01092-0

**Published:** 2021-07-16

**Authors:** Sonia Jaramillo, Richard F. Schlenk

**Affiliations:** 1 Department of Hematology, Oncology, and Rheumatology at Heidelberg University Hospital, University of Heidelberg, Heidelberg, Germany; 2 NCT-Trial Center, NCT Heidelberg, DKFZ and Heidelberg University Hospital, Im Neuenheimer Feld 130.3, 69120 Heidelberg, Germany

**Keywords:** Acute myeloid leukemia, Consolidation therapy, High-dose cytarabine, Midostaurin, Venetoclax, Gemtuzumab ozogamicin, VYXEOS, Ivosidenib, Enasidenib, Glasdegib, CC-486

## Abstract

**Purpose of Review:**

Until recently, improvement in terms of survival for patients with acute myeloid leukemia (AML) was achieved mostly in younger patients with dose intensification of conventional chemotherapy and a broadening use of allogeneic hematopoietic cell transplantation (allo-HCT) whereas the results remained dismal and very stable in patients older than 60 years. The current review highlights the recent developments in standard intensive post-remission chemotherapy, evidence for the use of recently approved agents, and discusses the relevance of measurable residual disease (MRD) measurement in treatment adaptation.

**Recent Findings:**

Current approvals of midostaurin, venetoclax, gemtuzumab ozogamicin, VYXEOS, ivosidenib, enasidenib, glasdegib, and CC-486 have changed the structure, aim, and schedule of consolidation therapy, and new, well-tolerated agents are being evaluated as maintenance therapies. Furthermore, MRD assessment has been implemented to guide the duration and type of consolidation and maintenance therapy as well as indicate the optimal timing of allo-HCT.

**Summary:**

Novel therapies have changed the structure and perspective of post-remission therapy in AML for both young and elderly patients. In addition, MRD assessment could guide the type, duration, and intensity of consolidation and maintenance therapy.

## Introduction

Acute myeloid leukemia (AML) is an aggressive, genetically heterogeneous hematological malignancy with still a poor prognosis [1, 2] characterized by the accumulation of somatically acquired genetic changes in hematopoietic progenitor cells, which leads to alterations in normal mechanisms of cell proliferation, and differentiation. It is currently considered to be the most common acute leukemia in adults with an incidence ranging from 2.0 in Korea [3] to 4.3 in the United States [4] per 100,000 men and women per year with a median age at diagnosis ranging in western countries between 65 and 72 years [5–7]. According to the National Cancer Institute’s surveillance program in the USA, the incidence of AML increases with age from 2 to 3 per 100,000 in young adults to 13 to 15 per 100,000 in the seventh and eighth decades of life [6]. Until the year 2000, improvements in survival were achieved mostly in younger patients (< 60 years) with dose intensification of conventional chemotherapy and a broadening use of allogeneic hematopoietic cell transplantation (HCT) whereas the results remained dismal and very stable in patients with an age ≥ 60 years [8]. Thus, currently curative treatment approaches were available only for a minority of AML patients. However, with the introduction of new treatment approaches, durable remissions are inducible nowadays also in older and even frail patient populations [9•, 10••, 11]. Therefore, the well-established ELN risk categorization [12•] with its risk adapted recommendations on intensity and type of consolidation therapy counterbalancing higher risk of relapse by treatment strategies [13], including allo-HCT, has to be revisited in light of recent approvals of several promising new drugs in AML.

The primary objective of the current review is to highlight the recent developments in standard intensive post-remission chemotherapy, evidence for the use of recently approved agents during consolidation therapy, and discuss whether and how measurable residual disease (MRD) could serve as a tool for continuous treatment adaptation.

## Intensive Chemotherapy

The concept of intensive post-remission chemotherapy in AML is based on the observation that (i) despite achievement of a first complete remission (CR) by intensive induction therapy virtually, all patients relapse in the absence of further treatment [14], (ii) randomized studies of young patients with AML showed that intensive post-remission chemotherapy is superior to prolonged low-dose maintenance therapy [15], and (iii) four repeated cycles of high-dose cytarabine (HiDAC) (3 g/m^2^, bid, days 1, 3, 5) are superior to intermediate- (400 mg/m^2^ cont. days 1–5) or standard-dose cytarabine (100 mg/m^2^ cont. days 1–5) with respect to relapse-free survival (RFS) and overall survival (OS) [15]. Furthermore, this concept is currently reinforced by recent findings in older patients with newly diagnosed *NPM1*-mutated [16], *IDH1*-mutated [17], and *IDH2*-mutated [11] AML responding well to a combination of venetoclax and azacitidine as well as single-agent ivosidenib or enasidenib, respectively, and receiving an undetermined duration of consolidation/maintenance therapy.

The current ELN 2017 guidelines recommend a consolidation regimen with cytarabine as monotherapy given bidaily (bid) on days 1, 3, and 5 [12•]. This recommendation is based on several clinical studies (for details, see Schlenk et al. [18]). The authors of the ELN 2017 recommendations stated that there is no convincing evidence available that favors HiDAC 3.0 g/m^2^ over intermediate-dose levels at 1.0–1.5 g/m^2^, with or without the addition of an anthracycline [12•, 19]. In contrast, the 2019 version of the NCCN guidelines considers the results of the two above mentioned studies in younger patients (< 60 years) with CBF AML and favorable-risk patients with negative MRD by recommending HiDAC 3.0 g/m^2^ bid. Similar to the ELN 2010 risk stratification, low risk AML is defined as normal karyotype with NPM1 mutation and absence of FLT3 mutation, isolated biallelic CEBPA, or core binding factor AML [20, 21].

Concerning the therapy schedule, the NCCN recommends, based on the findings of a cohort study performed within the AMLSG 07-04 study, to administer HiDAC on days 1, 2, and 3 as well as pegfilgrastim after chemotherapy in younger patients with AML in first complete remission [22, 23]. This last study showed that patients treated with HiDAC on days 1, 2, and 3 (n = 392) had a shorter hematologic recovery with white blood cells > 1.0 G/l and neutrophils > 0.5 G/l with in median 4 days shorter compared to HiDAC on days 1, 3, and 5 (P < .0001, each) and further reduced by 2 days (P < .0001) by pegfilgrastim administered on day 8, with significantly reduced rates of infections (P < .0001) and pegfilgrastim (P = .002). In addition, days in hospital and platelet transfusions were significantly reduced by HiDAC on days 1, 2, and 3 without affecting survival which was confirmed in a recent publication [24].

Furthermore, the post-remission therapy in the ALFA-0701 study for middle aged and older patients (age 50–70 years) used with two cycles of a combination with daunorubicin (60 mg/m^2^, day 1 first cycle and days 1–2 second cycle) and cytarabine (1 g/m^2^, bid, days 1–4) is also included for CD33 positive AML in the NCCN guidelines with GO (3 mg/m^2^, day 1) and is discussed further in the GO paragraph of the article [21, 25, 26].

Regarding the number of therapy cycles, a recent study included patients with intermediate- or good-risk AML after two cycles of induction therapy with daunorubicin, cytarabine, and gemtuzumab and explored different regimens of postinduction therapy. After a remission control, 1017 patients were randomized to one cycle of amsacrine, etoposide, and cytarabine or to a second cycle with mitoxantrone and cytarabine (n = 120). After a study amendment, patients were randomized to one or two cycles of consolidation therapy with HDAC (n = 897). Cumulative incidence of relapse and RFS at 5 years were improved in patients treated with two consolidation cycles compared to one (50% vs. 58%, P = .02 and 43% vs. 36%, P = .03, respectively). Interestingly, the impact on RFS was only significant when the second cycle was with HDAC [27].

## Midostaurin

Midostaurin (N-benzoyl-staurosporine) is an indocarbazol and was first developed as a protein kinase C inhibitor. During its development, it was found to be active against VEGFR-2, PDGFR, FGFR, c-Kit, and FLT3. Midostaurin leads to apoptotic cell death in *FLT3*-mutated AML cell lines, while it induces cell cycle arrest in *FLT3*-wildtype cell lines [28, 29]. The approval of midostaurin as adjunct to intensive chemotherapy in AML with activating *FLT3* mutations was based on the international multicenter randomized double-blinded phase-III RATIFY trial (n = 717) that investigated the efficacy of midostaurin versus placebo, during induction and consolidation therapy with conventional chemotherapy in young adults (18–59 years) with *FLT3*-mutated AML, and found an improvement in OS for patients randomized to midostaurin versus placebo (median OS increased from 26 to 75 months and 4-year OS increased from 44 to 51%) [28]. The FDA label includes midostaurin 50 mg twice daily on days 8–21 of each cycle of consolidation with HiDAC. However, the evaluation of 12-month maintenance therapy after completion of consolidation therapy was not based on a second randomization nor was the post-allogeneic stem cell transplantation (allo-HCT) maintenance with midostaurin. Thus, the maintenance therapy with midostaurin is not explicitly recommended on the label. The approval granted by the European Medicines Agency (EMA) included single-agent maintenance therapy for *FLT3*-mutated AML patients in CR and although the studied patient population was younger than 60 years, both agencies approved midostaurin without an upper age limit. Regarding maintenance therapy after allo-HCT, the single arm AMLSG 16-10 phase-II trial provided robust data about the safety and efficacy of midostaurin in younger (18–60 years) and older (60 to 70 years) patients with *FLT3-ITD* positive AML. The tolerance of maintenance therapy after allogenic HCT was insufficient with less than 50% of the patients finishing the 1 year intended maintenance therapy. The median time of maintenance therapy was 9 months. However, the comparison to historical controls showed beneficial effect of midostaurin regarding event free survival (EFS) (P = 0.01) and OS (P = 0.02) [29, 30].

An exploratory analysis of the RATIFY study also helped clarify the efficacy of midostaurin in patients receiving allo-HCT. This study found that patients who underwent allo-HCT after achieving a first CR even beyond day 60 of induction therapy and received therapy with midostaurin had a significantly lower cumulative incidence of relapse (CIR) than patients who did not receive midostaurin (p = 0.02) [31]. Taken together, there are no randomized data that provide clear benefit of midostaurin in combination with consolidation chemotherapy or as maintenance therapy. However, these data indicate that the addition of midostaurin to first induction therapy is of extreme importance to induce the observed beneficial effect including the reduced relapse rate in patients after allogeneic HCT in first CR. This seems to be particularly true for ELN high-risk patients as those patients benefitted most from midostaurin before allo-HCT [32] whereas patients with ELN intermediate and low risk AML had better overall survival compared to patients in the placebo arm of the study but no additional benefit of allo-HCT [32].

## Gemtuzumab Ozogamicin

Gemtuzumab ozogamicin (GO) is a humanized immunoglobulin G4 antibody (hP67.6) directed against CD33 and conjugated via a hydrolyzable linker to the DNA toxin calicheamicin. GO/CD33 complexes are internalized into lysosomes, releasing calicheamicin and promoting single- and double-strand breaks and cellular death. After initially accelerated FDA approval in 2000 for the treatment of CD33-positive-AML aged ≥ 60 years in first relapse, GO was withdrawn from the market in June 2010 due to negative results of the phase 3 Southwest Oncology Group (SWOG) study S0106 showing significantly higher induction mortality without improving CR or relapse-free survival [33, 34]. Based on the results of the ALFA-0701 study in newly diagnosed patients [25], the AML-19 study in patients with newly diagnosed AML unsuitable for intensive chemotherapy [35], and the MyloFrance-1 study in relapsed/refractory AML [36], GO was reapproved by the Food and Drug Administration (FDA) for the treatment of newly diagnosed CD33-positive AML in adults and treatment of relapsed or refractory CD33-positive AML in adults and in pediatric patients 2 years and older. The approval granted by the European Medicines Agency (EMA) included only patients aged 15 years and older with previously untreated, de novo, CD33-positive-AML. Both approvals included besides fractionated dosing (3 mg/m^2^, days 1, 4, 7) in induction therapy also the addition of GO (3 mg/m^2^, day 1) to consolidation therapy with daunorubicin and cytarabine [25]. Although CR with or without platelet recovery and early deaths were similar, patients in the GO arm had significantly improved median event-free (19.6 vs. 11.9 months; P = .00018) and OS (34 vs. 19.2 months; P = .046) [25]. However, OS in the GO arm did not reach statistical significance in the final analysis of the ALFA-0701 trial with a median follow-up of 47.6 months [26]. Nonetheless, the longer OS trend in the GO arm observed in ALFA-0701 is consistent with the results demonstrated in an individual patient data meta-analysis that showed a significant improvement in OS of patients treated with GO [37]. Regarding post-remission therapy, two trials assessed GO on a randomized basis, where no significant impact on survival was observed (for details, see Burnett et al. and Löwenberg et al. [38, 39]). In a recently published randomized trial, the AMLSG 09-09 study, 588 adult patients with newly diagnosed *NPM1*-mutated AML were randomly assigned to the standard arm (n = 296) with induction therapy consisting of idarubicin (12 mg/m^2^ on days 1, 3, and 5 or days 1 and 3 in patients age > 60 years or in the 2nd cycle), cytarabine (100 mg/m^2^ continuously IV on days 1 to 7), etoposide (100 mg/m^2^ IV on days 1 to 3 or days 1 and 3 in patients age > 60 years or in the 2nd cycle), and all-trans-retinoic acid (ATRA) (45 mg/m^2^ orally on days 6 to 8 and 15 mg/m^2^ on days 9 to 21) and the GO arm (n = 292) with GO as adjunct to standard arm therapy at day one in a dosage of 3 mg/m^2^. The early primary endpoint EFS was not significantly different (hazard ratio (HR), 0.83; 95% CI, 0.65 to 1.04; P = .10), mainly due to a significantly higher infection triggered early death rate during induction therapy with 10.3% in the GO arm and 5.7% in the standard arm (P = .05). Although the early primary endpoint EFS was not met, the cumulative incidence of relapse (CIR) in patients achieving a CR or CR with incomplete hematologic recovery (CRi) was significantly and more important clinically relevantly reduced in the GO arm compared with the standard arm (P = .005), with no difference in the cumulative incidence of death (P = .80). Patients who achieved a CR or CRi after induction therapy received consolidation therapy. This consisted of 3 cycles of high-dose cytarabine plus ATRA. Cytarabine was dosed differently according to patient’s age (18–60 years, 3 g/m^2^ every 12 h on days 1 to 3; and > 60 years, 1 g/m^2^ every 12 h on days 1 to 3). ATRA was administered orally (15 mg/m^2^/day PO on days 4 to 21), and pegfilgrastim (6 mg subcutaneously) was given on day 8 in both study arms. Patients in the GO arm received GO (3 mg/m^2^ IV on day 1) during the first consolidation therapy [40].

In a companion study evaluating *NPM1* MRD during the trial, overall, the addition of GO reduced significantly the MRD levels at all time-points compared to the standard arm. This study clearly demonstrated the value of consolidation therapy with high-dose cytarabine, since *NPM1* MRD was significantly and sequentially reduced during the 3 consolidation cycles in the standard- as well as in the GO-arm of the study, thus strongly supporting the concept of intensive post-remission therapy [41••]. However, despite achieving *NPM1* MRD negativity after consolidation therapy, still one-quarter of the patients relapse within 4 years [41••]. This indicates that further efforts have to be invested in refining MRD assessment and finding its relevance in particular parallel to the evaluation of maintenance therapy as discussed below.

## Enasidenib and Ivosidenib

Somatic mutations within the conserved active site of isocitrate dehydrogenase (*IDH*) 1 and 2 have been found in ~8% and ~12% of AML cases, respectively [42–45]. *IDH1* and *IDH2* mutations produce an oncometabolite, 2-hydroxyglutarate (2-HG), which leads to DNA and histone hypermethylation, impaired hematopoietic differentiation, epigenetic alterations, and impaired hematopoietic differentiation [46–48]. Enasidenib is an oral, selective inhibitor of mutated-*IDH2* enzymes, approved on by the FDA for the treatment of adult patients with relapsed or refractory *IDH2*-mutated AML. Its approval was based on the results of a phase-1/2 trial including 176 patients [49]. The overall response rate was 40.3% with 65% being CRs. During a follow-up of in median 7.7 months, 56 patients of 71 responding patients progressed or relapsed.

Ivosidenib is an oral small molecule inhibitor of mutated *IDH1* approved by the FDA for the treatment of adult patients with relapsed or refractory AML with a susceptible *IDH1* mutation. The approval was based on results of an open-label, phase 1 dose-escalation and dose-expansion study with n = 258 adult patients with *IDH1-*mutated AML. In the primary efficacy population consisted of 125 patients, overall response rate (ORR) ranged from 39.1% (95% CI, 31.9–46.7%) in relapsed/refractory AML to 55.9% (95% CI, 37.9–72.8%) in newly diagnosed AML. Patients who had received one prior regimen had a CR + CR with partial hematologic recovery (CRh) rate of 46.0% (95% CI 31.8, 60.7) [50•].

A recent publication reported the response of 34 newly diagnosed AML patients ineligible for standard therapy treated with 500 mg Ivosidenib monotherapy. Overall response rate (ORR) was 54.5% (95% CI, 36.4–71.9%), CR/CRh rate was 42.4% (95% confidence interval [CI], 25.5% to 60.8%), and median duration of CR + CRh with median follow-up of 23.5 months was not reached. Of note, median age was 76.5 years, and the therapy was well-tolerated [17].

For both IDH inhibitors, treatment strategies with the aim of maintaining response, which is the classical goal of consolidation and maintenance therapy, are urgently needed. An ongoing trial (NCT02632708) is assessing the safety and the efficacy of ivosidenib and enasidenib in combination with induction and consolidation chemotherapy in patients with de novo *IDH1*- and *IDH2*-mutated AML. An interim analysis reported among 60 efficacy-evaluable ivosidenib-treated patients that CR, CRi, or CRp was achieved in 77%. Of the 91 efficacy-evaluable enasidenib-treated patients, a response of CR, CRi, or CRp was achieved in 74% of patients [51]. Furthermore, patients with a best overall response of CR/CRi/CRp receiving ivosidenib had *IDH1* mutation clearance by digital polymerase chain reaction in 39% of the cases as well as an *IDH2* mutation clearance in 23% of the cases receiving enasidenib [51].

Another ongoing study (NCT03839771), a phase III multicenter, double blind, randomized, placebo-controlled study, is also evaluating the efficacy of ivosidenib and enasidenib in combination with intensive induction therapy, consolidation therapy, and maintenance therapy in patients with AML or myelodysplastic syndrome (MDS) with excess blasts-2 with an *IDH1* or *IDH2* mutation.

## VYXEOS (CPX-351)

VYXEOS is a dual-drug liposomal encapsulation of cytarabine/daunorubicin in a 5:1 molar ratio. It was approved based on a randomized study that compared VYXEOS to standard induction and consolidation therapy in older patients (60–75 years). Patients were included if one of the following constellations were present: therapy associated AML, history of MDS, AML with a history of chronic myelo-monocytic leukemia (CMMoL), or de novo AML with myelodysplasia related cytogenetic changes [52]. The study showed that patients receiving CPX-351 achieved a higher CR rate (47.7% vs. 33.3%, P = .016) and OS (median, 9.56 vs. 5.95 months; P = .005) compared to standard induction and consolidation therapy [52].

A recent phase 3 study randomized 309 older patients aged 60–75 years with de novo high-risk/secondary AML to received 1–2 induction cycles with CPX-351 (n = 153) or “7 + 3” (n = 151) chemotherapy. Patients achieving CR/complete remission with incomplete count recovery (CRi) received up to 2 consolidation cycles with CPX-351 or lower-dose cytarabine combined with an anthracycline, respectively. Complete remission or CRi was achieved by 48% of the patients treated with CPX-351 induction, and 33% of the patients given “7 + 3” induction [53••]. Furthermore, no clinically meaningful differences between treatment arms in the frequencies of treatment-emergent adverse events were seen. VYXEOS consolidation was administered to 49 (32%) patients, and low-dose cytarabine + anthracycline was administered to 32 (62%) patients. Median OS was prolonged among patients who received VYXEOS through induction and consolidation versus “7 + 3” and low-dose cytarabine + anthracycline (25.43 vs. 8.53 months, respectively; HR = 0.44 [95% CI: 0.25–0.77] [53••]. Thus, it can be concluded that in older adults with newly diagnosed high-risk/sAML, who received both induction and consolidation with VYXEOS, OS was significantly improved versus conventional induction and consolidation therapy.

## Patients Unfit for Intensive Post-Remission Therapy

Elderly patients are more likely to have comorbidities, decreased performance status, and poorer cytogenetic risk [5]. These factors contribute to poorer prognoses and poorer tolerance to intensive therapeutic regimens. Other less intensive treatment options such as the hypomethylating agents azacitidine and decitabine have become the backbone for the majority of therapy combinations in elderly patients [54, 55].

Targeted therapy such as GO has been shown to be effective in older adults (> 60 years), unfit for intensive therapy. In a randomized study, low-dose GO (6 mg/m^2^ on day 1 and 3 mg/m^2^ on day 8 of induction, followed by monthly doses of 2 mg/m^2^ as consolidation) (n = 118) was compared to BSC (n = 119), which resulted in a 1-year OS rate of 24.3% (95% CI, 16.9 to 32.4) with GO and 9.7% (95% CI, 5.1 to 15.9) with BSC. No increase toxicity was observed with GO [35].

Ventoclax, a potent BCL-2 inhibitor in a single-agent phase II trial, demonstrated an overall response rate of 19% (6/32) being 6% (3/32) CR in a very advanced AML population with a median age of 71 years [56]. Based on the synergistic effect of venetoclax and hypomethylating agents observed in preclinical ex vivo models of AML cells in elderly patients unfit for standard induction, in a phase 1b dose-escalation study, 145 elderly patients (> 65 years) with de novo AML and ineligible for intensive chemotherapy were treated with venetoclax at 400, 800, or 1200 mg daily in combination with either decitabine (20 mg/m^2^, days 1–5) or azacitidine (75 mg/m^2^, days 1–7). After a median follow-up time of 8.9 months, CR and CRi rate was of 73% in the venetoclax 400 mg and hypomethylating agent cohort [57].

In a recent study, 431 elderly unfit patients with AML, ineligible for standard induction therapy, were treated with azacitidine plus either venetoclax or placebo. The median overall survival was higher in the azacitidine–venetoclax group with 14.7 versus 9.6 months in the placebo group (HR 0.66; 95% confidence interval, 0.52 to 0.85; P < 0.001). The incidence of complete remission was also higher in the experimental arm 36.7% vs. 17.9%, respectively (P < 0.001) [10••].

An international phase 3 randomized double-blind placebo-controlled trial treated 211 elderly unfit patients with AML with either venetoclax (n = 143) or placebo (n = 68) as adjunct to low-dose cytarabine (LDAC). The median follow-up was 12 months; however, a significant OS difference was seen after an additional 6-month follow-up. The median OS was 8.4 months in the venetoclax arm and 4.1 months in the control arm (HR, 0.70; 95% CI, 0.50–0.98; *P* = .04). CR and CRi were 48% (95% CI, 39–56%) in the venetoclax arm versus 13% (95% CI, 6–24%) in the placebo arm [58•].

Thus, it is safe to say that venetoclax plus hypomethylating agent or LDAC demonstrated a meaningful improvement in remission rate and OS compared to chemotherapy alone.

Currently, two ongoing clinical trials are exploring venetoclax with different non-intensive chemotherapy backbones in patients with de novo AML. In the phase 2 trial (NCT03466294) enrolling elderly patients, venetoclax is given in combination with 5-AZA. Patients achieving MRD negativity after induction treatment receive a maintenance therapy with venetoclax alone. In the phase 1b study (NCT03709758) designed for younger patients, venetoclax is combined with intensive induction and consolidation chemotherapy. Both trials will add more data on the role of venetoclax in the upfront treatment of patients with AML. Another recent study evaluated venetoclax in combination with fludarabine, cytarabine, and granulocyte colony stimulating factors in patients with newly diagnosed and relapse/refractory AML. Overall response rate was 89% in newly diagnosed AML and 1-year OS was 92% [59, 60]. Final reports of these studies are expected to generate changes of the therapeutic paradigms in AML. Other studies presented at the virtual 62nd American Society of Hematology (ASH) Annual Meeting proposed different therapy regimens and combination therapy partners of venetoclax. One of the most promising combinations was shown the Blast MRD AML-2 study, where venetoclax was combined with azacytidine and pembrolizumab a PD-1 inhibitor [61]. Also, in unfit patients, different combination partners for venetoclax, e.g., pevonedistat, a first in class inhibitor of Nedd8, and cadribine, a purine analogue, were presented [62, 63].

## Glasdegib

Glasdegib is a selective, oral, small-molecule inhibitor of the hedgehog receptor of smoothened (SMO). In a serial transplantation mouse model, in vivo treatment of AML cells with glasdegib attenuated the leukemia-initiation potential [64]. In two phase I trials in adult patients with refractory myeloid malignancies, glasdegib was well-tolerated and showed a response rate as high as 49% [65, 66]. Glasdegib was approved by the FDA in combination with low-dose cytarabine for unfit AML patients following a randomized phase II study that compared low-dose cytarabine with (n = 88) or without (n = 44) glasdegib. The experimental arm demonstrated a statistically significant improved CR rate (15% versus 2.3%) and OS (8.3 months versus 4.3 months) (HR, 0.46 [80% CI, 0.35–0.62], P = 0.0002) [67]. Inline, encouraging results of a first interim analysis of the open-label, multicenter, phase 1b BRIGHT MDS & AML 1012 (NCT02367456) trial reported, after a median follow-up time of 7.8 months, a CR of 20.0% (n = 6) in patients unable to receive intensive chemotherapy or age ≥ 75 years with AML that received treatment with glasdegib and azacytidine [68]. Taken together, all recently approved therapies significantly improve response to therapy; however, their role in the consolidation scenario is still unclear and further trials are urgently needed.

## Maintenance Therapy

In the HOVON97 trial, 116 AML or MDS patients age 60 years or older in CR/CRi were randomized to 5-day azacitidine and observation. A significant difference at 36 months was reported in terms of disease-free survival (DFS) in the 5-day azacitidine arm (32 vs. 16% P = .04) [69]. An ongoing phase 3 trial randomized 149 older (> 60 years) AML patients in first remission to azacitidine or observation after completing two courses of intensive induction and one cycle of intermediate-dose cytarabine consolidation. An interim analysis of 54 patients after an observation period of 15 months showed that in patients older than 73 years, there was a significant benefit of maintenance therapy with azacytidine in terms of DFS and OS compared to best supportive care (P < 0.0001 and P = 0.005, respectively) [70]. A recent clinical trial, the QUAZAR study, a phase III international, randomized, double-blind, placebo-controlled study, evaluated CC-486, an oral well-tolerated hypomethylating agent, as maintenance therapy in elderly patients above the age of 55 years with intermediate or high-risk AML after achieving first CR or CRi after intensive induction therapy and ineligible for allo-HCT. The maintenance therapy was intended until death, relapse, or intolerable toxicity. According to a previous study report, at a median follow-up of 41.2 months, CC-486 lead to a significant improvement of OS compared to placebo arm (24.7 months vs. 14.8 months, respectively, HR 0.69; [95% CI 0.55, 0.86], P = 0.0009). Relapse-free survival was also significantly prolonged: median RFS was 10.2 months in the CC-486 arm, compared to 4.8 months in the placebo arm (P = 0.0001; HR 0.65 [95% CI 0.52, 0.81]). The most frequently reported adverse events (AEs) were grade 1 or 2 nausea, vomiting, and diarrhea. The most common grade 3–4 AEs were neutropenia, anemia, and thrombocytopenia. Based on these results, the FDA granted an approval of CC-486 in September 2020 for patients in CR/CRi unfit for allo-HCT or intensive consolidation therapy after intensive induction therapy, making it the first treatment used in the maintenance setting to provide statistically significant and clinically meaningful improvement in OS and RFS after first CR [71••].

## Future Direction of the Consolidation Therapy

**Fig. 1:**
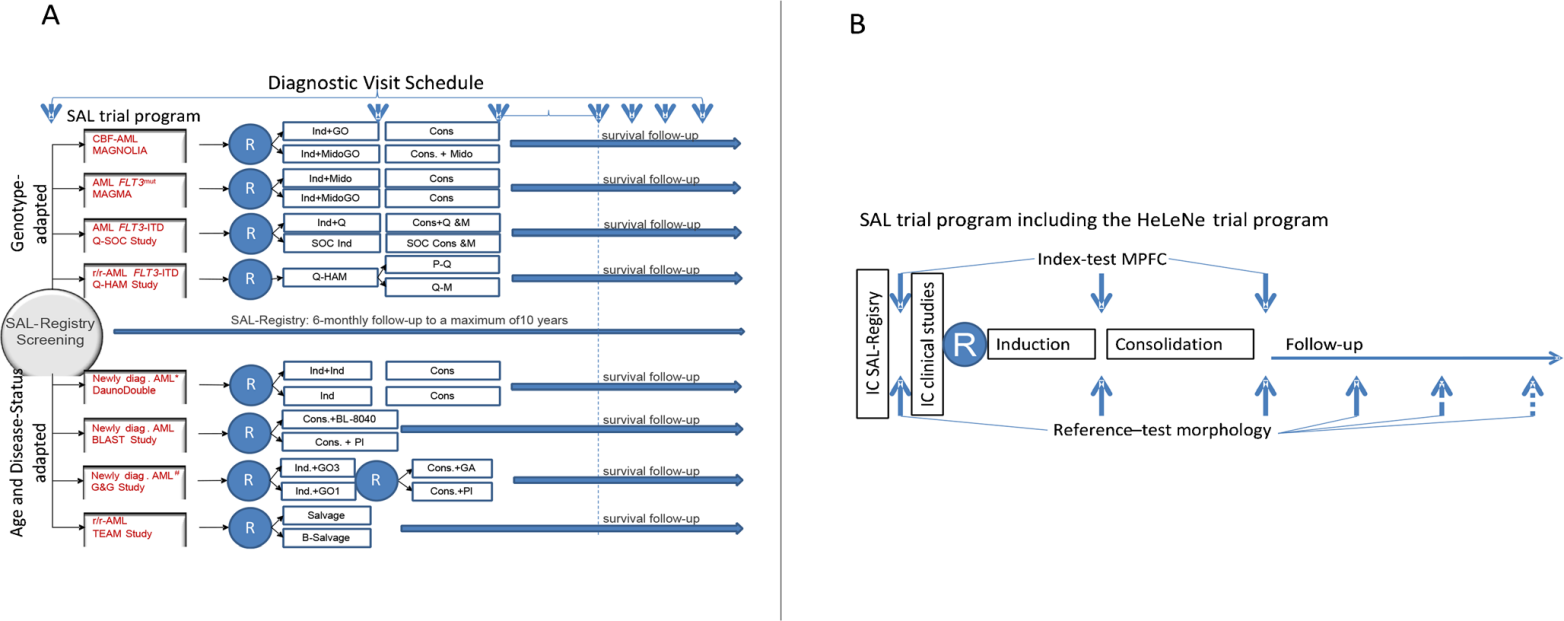
Prospective evaluation of measurable residual disease in intensively treated patients with acute myeloid leukemia (AML) as surrogate endpoint for survival (PERDAM Project). **A** Interventional prospective randomized trials of the SAL. **B** Diagnostic meta-study on MRD. *age 18–65, #age > 60 years. Abbreviations: B, Bortecomib; BL-8040, CXCR4 inhibitor; CBF-AML, core-binding-factor acute myeloid leukemia; Cons, consolidation; diag., diagnosed; GA, glasdegib; GO, gemtuzumab ozogamicin; Ind, induction; Mido, midostaurin; Pl, placebo; r/r-AML, refractory/relapsed AML; SAL, Study Alliance Leukemia; Q, quizartinib; HAM, high-dose cytarabine and mitoxantrone; P, preemptive; M, maintenance; SOC, standard of care; HLP, Heidelberg Leukemia Program; IC, informed consent; MPFC, multiparameter flow cytometry

Response to AML treatment is highly variable and despite well stablished risk classification, treatment response for some patients is still unpredictable. Recent studies suggest that morphological CR alone should no longer be considered as the main criterion to evaluate response to treatment because light microscopy has limited sensitivity and is hampered by inter-observer variability [72]. Several studies have proposed MRD as a powerful predictor of clinical outcome. There are currently several diagnostic tools to assess residual disease [73]. It can be measured by polymerase chain reaction (PCR) [74, 75], multiparameter flow cytometry (MPFC) [76–78], and next-generation sequencing (NGS). Consistently across all methods, the prognostic values of MRD assessment at the end of induction therapy and after completion of consolidation therapy have been shown [79–81]. Recent studies found a significant and strong correlation between *NPM1* MRD-positivity immediately before allo-HCT, and adverse outcome regarding OS and DFS [41••, 82, 83]. Furthermore, a recent study showed that MRD positivity was significantly associated with worse OS (HR, 1.85; p < .0001) and RFS (HR, 2.04; p < .0001) [84]. Thus, it is safe to say that MRD assessment can influence treatment decisions in individual patients regarding the choice of consolidation therapy. Unfortunately, more than 50% of relapses are not predicted by molecular MRD assessment and occur in the MRD-negative groups [85, 86]. However, so far, no prospective confirmatory evaluation has been performed within randomized clinical trials defining the MRD assessment sensitivity and specificity of alternative methods such as MPFC. With the number of new compounds that can potentially be tested in the clinic, and the need to reach conclusions about the safety and efficacy of new treatments as quickly as possible, the search for early clinical endpoints and biomarkers that can be used as surrogate endpoints for long-term clinical endpoints has emerged as an urgent need. To improve and individualize the definition of disease burden and treatment response, MRD assessment by MPFC or molecular techniques should become standard in every clinical trial in AML. The Study Alliance Leukemia (SAL) group started a diagnostic meta-study on MRD, enrolling all patients with AML participating in interventional prospective randomized trials of the SAL (Fig. 1A), including interventional prospective randomized trials initiated from the University Hospital Heidelberg. In all evaluable patients, initial diagnostic flow cytometric analysis and assessment of MRD after induction (newly diagnosed AML) or salvage chemotherapy (relapse/refractory AML) and at the end of consolidation therapy are intended (Fig. 1B). Within our study, we want to evaluate the performance from MRD by MPFC as an early biomarker surrogate for survival endpoints. The direct comparison of quantified MRD evaluated by MPFC, by real-time quantitative polymerase chain reaction (RQ-PCR) [87] for gene mutations and the gene fusions, and by NGS within prospectively randomized trials offers the unique opportunity of a pivotal reference cross validation of the different methods for recent and future studies. Based on the still poor outcome of AML in general, there is an increasing public demand that new, promising drugs are approved for therapy as rapidly as possible. Furthermore, MRD assessment could guide the duration of consolidation and maintenance therapy as well as clarify the necessity of further therapies to prevent an early relapse.

## Conclusion

Recent therapy approvals have changed the structure and perspective of post-remission therapy in AML for young as well as elderly patients. In addition, MRD assessment could guide the type, duration, and intensity of consolidation and maintenance therapy.
